# Incidence age is bimodal for myalgic encephalomyelitis/chronic fatigue syndrome, with higher severity burden for early onset disease

**DOI:** 10.1093/oxfimm/iqag007

**Published:** 2026-03-17

**Authors:** Simon J McGrath, Charles B Hillier, Joshua J Dibble, Trude Schei, Arild Angelsen, Audrey A Ryback

**Affiliations:** c/o Action for ME, Unit 2.2 Streamline, 436-441 Paintworks, Bristol, BS4 3AS, United Kingdom; c/o Action for ME, Unit 2.2 Streamline, 436-441 Paintworks, Bristol, BS4 3AS, United Kingdom; MRC Human Genetics Unit, Institute of Genetics and Cancer, University of Edinburgh, Edinburgh, Scotland, EH4 2XU, United Kingdom; Norwegian ME Association, Nedre Slottsgate 4 M, 0157, Oslo, Norway; School of Economics and Business, Norwegian University of Life Sciences (NMBU), PO Box 5003, 1432, Ås, Norway; MRC Human Genetics Unit, Institute of Genetics and Cancer, University of Edinburgh, Edinburgh, Scotland, EH4 2XU, United Kingdom

**Keywords:** myalgic encephalomyelitis, myalgic encephalomyelitis / chronic fatigue syndrome, chronic fatigue syndrome, age at onset, bimodal onset age, disease severity, infectious triggers, glandular fever, infectious mononucleosis, epidemiology

## Abstract

Myalgic Encephalomyelitis, or Chronic Fatigue Syndrome (ME/CFS), is a disease of uncertain origin. Studies of Norwegian health records have suggested that ME/CFS incidence across age groups is bimodal—a characteristic that could provide insight into the aetiology of the disease. Here, we analysed survey data from over 9000 respondents with ME/CFS from 10 European countries, and observe an early onset peak with a mean of 16.0 years old (standard deviation [SD]: 4.3) and a late onset peak at 36.6 years old (SD: 10.5). Statistical support for multimodal onset age was evident in 7 of the 10 countries examined. Infection as a trigger for ME/CFS was 10 percentage points higher among early compared to late onset disease (*p* = 2.1 × 10^−13^). Early onset ME/CFS was associated with greater odds of being severely or very severely affected (OR = 2.15, 95% CI [1.84–2.51], *p* < 2 × 10^−16^). Those with first degree relatives with ME/CFS had greater odds of early than late onset ME/CFS (OR = 1.43, 95% CI [1.25–1.63], *p* = 4.4 × 10^−07^). We further validated our findings in a UK dataset where we replicated bimodal onset age and observed significantly greater odds of glandular fever/infectious mononucleosis as a trigger in early onset cases (OR = 2.32, 95% CI [1.99–2.71], *p* = 2.4 × 10^−24^). Our findings suggest that incidence of ME/CFS peaks in adolescence and in early middle-age and that early onset ME/CFS is more common in those with affected relatives, more often triggered by infection, and associated with more severe disease.

## Introduction

Myalgic Encephalomyelitis/Chronic Fatigue Syndrome (ME/CFS) is a highly disabling disease of uncertain aetiology and under-researched mechanisms. ME/CFS has a high prevalence of 0.4%–0.6% [1, 2] reaching as high as 0.75% in paediatric populations [3]. The true prevalence of ME/CFS remains uncertain, particularly in the wake of the SARS-CoV-2 pandemic, with a large proportion of Long Covid patients also meeting ME/CFS diagnostic criteria [4, 5]. Full recovery from ME/CFS is rare in adults, at around 5% [6, 7]. ME/CFS is associated with very poor health-related quality of life [8]. Large-scale survey data suggests that 13%–18% of people with ME/CFS are severe (mostly bedbound) or very severely affected (bedbound and in need of care), and more than half have moderate disease—many of whom are mostly housebound [9, 10]. There is a female preponderance with about four times as many females diagnosed with ME/CFS as males [2, 11, 12]. DecodeME, the largest genetic study of ME/CFS to date, has provided a recent scientific breakthrough with 8 genetic loci discovered that alter the odds of developing ME/CFS, linked primarily to innate immunity and the nervous system [10]. Prospective cohort studies have demonstrated that several acute infections, including Epstein-Barr virus/infectious mononucleosis, lead to ME/CFS in around 10% of cases followed up at 12 months [13, 14]. Infection is the most common trigger reported by people with ME/CFS: 49% of EMEA respondents included in this study reported infection prior to illness onset. 62.4% of DecodeME participants reported infection as the trigger for their illness, of which 27% reported infectious mononucleosis/glandular fever as the trigger [12].

The ages at which diseases manifest are often tied to their causes and pathomechanisms, and can help to identify disease subgroups [15]. For example, in autoimmune vitiligo, two incidence age peaks are associated with distinct genetic risk and clinical features. Childhood onset disease is associated with a genetic haplotype that confers extreme genetic risk [odds ratio [OR] > 8), not seen in individuals with late onset disease, which is associated with environmental triggers and concomitant autoimmune disease [16, 17]. Thus, in diseases with multi-modal onset age distributions, distinct aetiologies could underpin the elevated incidence for specific age groups.

Healthcare data from Norway suggests that ME/CFS incidence is not uniform across age groups. Bakken *et al*. (2014) studied 5809 individuals with an ICD-10 code for ‘post-viral and related fatigue syndromes’ (G93.3) in the Norwegian patient register which includes hospital and specialist outpatient clinic records between 2008–2012 for the entire population of Norway [18]. They found that G93.3 diagnoses were most frequent in age groups 10–19 and 30–39 years. Another study of individuals with an ME/CFS diagnosis in the same database between 2016–2018 observed the highest incidence in the 15–19 years age group followed by the 35–39 years age group [19]. A further study of the same population, adjusting for demographic confounders, found that the odds ratio of an ME/CFS diagnosis was highest in the 18–24 age group [11]. A study of age at CFS/systemic exertion intolerance disease (SEID) diagnosis using health records in Finland between 2002–2012 found that diagnoses were most common in the 25–30 years age groups, followed by 16–20 years, although the sample size was small (*n* = 327) [20]. However, these studies examined age at diagnosis, rather than age at onset, and did not estimate the parameters of these putative peaks in incidence. Age at diagnosis may be an inaccurate proxy for age at onset since ME/CFS diagnosis is often delayed. In one survey-based study in the UK, 22.1% of patients were diagnosed within 1–2 years, and 12.9% took more than 10 years [21]. The EMEA report found the average diagnostic delay was 5 years in the UK, 6 years in Norway and up to 12 years in other European countries [9]. Therefore, onset age estimates are likely to be less accurate in studies relying on age at diagnosis than age at symptom onset. Furthermore, studies from Norway and the UK have demonstrated that the incidence of G93.3 diagnoses is significantly lower in more deprived postcodes or households, suggesting that access to diagnosis is affected by socio-economic status [2, 11].

In this study we set out to explore whether this bimodal onset age pattern in ME/CFS is observed in other countries and could therefore provide clues to the aetiologies or risk factors for ME/CFS. We analysed data from a pan-European survey of people with ME/CFS from 2021 led by the Norwegian ME Association. They collected data from 11297 respondents and captured onset age and year as well as relevant factors, including the reported trigger for the illness, whether they had first degree relatives with ME/CFS, and how severely respondents were affected by ME/CFS. We further validated our findings in a subset of participants from DecodeME.

Our primary aims were to:

1. Test whether there was evidence for bimodal onset age of ME/CFS in Norway and other European countries; and,
2. Explore whether there were differences in phenotypes between early or late onset ME/CFS.

## Materials and methods

### Data sources and cleaning

#### EMEA

Data from the 2021 European ME Association (EMEA) survey was shared and re-analysed with permission from the Norwegian ME association who ran the survey [9]. The survey was promoted by national ME patient organisations on their websites, by e-mails, and on social media. Ethical review and approval were waived for the study due to it being an anonymous, online survey.

We selected countries that had an estimated coverage of >0.1% of the country’s ME/CFS population, assuming a 0.4% prevalence and a minimum of 200 responses. We removed any responses that did not report a year of birth and year of onset. Age at onset was derived from the responses to ‘Year of birth’ and ‘When did you become ill with ME?’. Trigger types were ascertained from the multiple-choice answer to: ‘Do you associate the start of your illness with a particular event?’. First-degree relatives were ascertained from answers to the question: ‘Do you have close relatives (children, parents or siblings) with ME?’.

#### DecodeME data

A subset of DecodeME participants were analysed for this study with data provided by the DecodeME team. Individuals were selected who reported a disease duration of 0.5–1 years, 1–3 years, 3–5 years or 5–10 years, and those reporting either ‘glandular fever/infectious mononucleosis’, ‘other infection’ or ‘no infection’ as a trigger for their illness. To generate the approximate age at onset, the midpoint of each duration bin (i.e. 0.75, 2, 4, or 7.5 years) was subtracted from the respondents’ age when they completed the survey. Approximate ages at onset were additionally rounded to the nearest integer to fit splines and to perform Hartigan’s Dip Tests.

### Statistical and data analysis

Plots were generated using ‘ggplot2’ (version 3.5.0) and maps were made using ‘rnaturalearth’ (version 1.0.1) [22] and ‘sf’ (1.0–15) [23] *R* packages (R version 4.2.2).

Splines were fitted using generalised additive models in ggplot2, using the ‘geom_smooth’ function, with counts of each age (in 1-year bins) as the predictor. Thin plate regression splines were fitted, and dimensions were set empirically.

To test whether there was statistical evidence in support of multi-modality, we performed a Hartigan’s Dip Test [24]. This calculates the maximum distance between the empirical data and its hypothetical closest unimodal distribution. The ‘diptest’ R package (version 0.77-1) was used to compute Hartigan’s dip statistic, and its *P*-value. *P*-values were generated by Monte Carlo simulation with 2000 replicates, at a significance level of alpha = 0.05. Gaussian mixture models were fitted with the ‘mixtools’ R package (version 2.0.0) [25] using ‘normalmixEM’ and specifying two components (distributions), with the following specifications:


normalmixEM (data=age_ill,k=2, maxit=1000, epsilon=1e−06, maxrestarts=2)

Its algorithm for normally distributed mixtures maximizes the conditional expected complete-data log-likelihood. Estimands for means, standard deviations, mixing proportions (lambda), and log-likelihoods were extracted from model outputs. Since there is a degree of stochasticity in how mixtools fits the Gaussian mixture models, each model was run 1000 times per country and the estimates from the model with the greatest log-likelihood were reported.

Comparisons between early and late onset groups were calculated from the probability of observing that datapoint under the first or second distribution, based on the estimates for early and late onset ME/CFS distributions using all of the data. This was calculated by dividing the likelihood of each datapoint under each distribution by the sum of the likelihood of each datapoint under both distributions. We summed these to generate probability-weighted estimates for the proportion of individuals belonging to early and late onset distributions.

Chi-squared tests of independence were performed using the chisq.test() function in base *R* (version 4.2.2 (2022-10-31)). In post hoc tests for individual categories, *P*-values for the individual categories were calculated from the standardised residuals and corrected for multiple testing by applying a Bonferroni correction [26].

To explore the association between early and late onset and severity, the following non-proportional odds ordinal logistic regression model was run using ‘vglm’ in the ‘VGAM’ R package (version 1.1.9) [27]:

Severity ∼ duration + probability_late

This produced odds ratios for the five possible comparisons in severity:

- Recovered **versus** >mild, mild, moderate, severe or very severe
- Recovered or >mild **versus** mild, moderate, severe or very severe
- Recovered, >mild or mild, **versus** moderate, severe or very severe
- Recovered, >mild, mild or moderate **versus** severe or very severe
- Recovered, >mild, mild, moderate or severe **versus** very severe.

We reported the odds ratio for recovered, >mild, mild or moderate to severe or very severe, between the early and late onset groups. The odds ratios for the other severity comparisons, and their associations with duration are reported in Supplementary Table 1.

The variance inflation factor was calculated using the formula 1/(1-*R*^2^), where *R*^2^ was obtained from correlating duration with assigned distribution:


duration∼probability_late

## Results

### Survey respondent demographics

**Figure 1:**
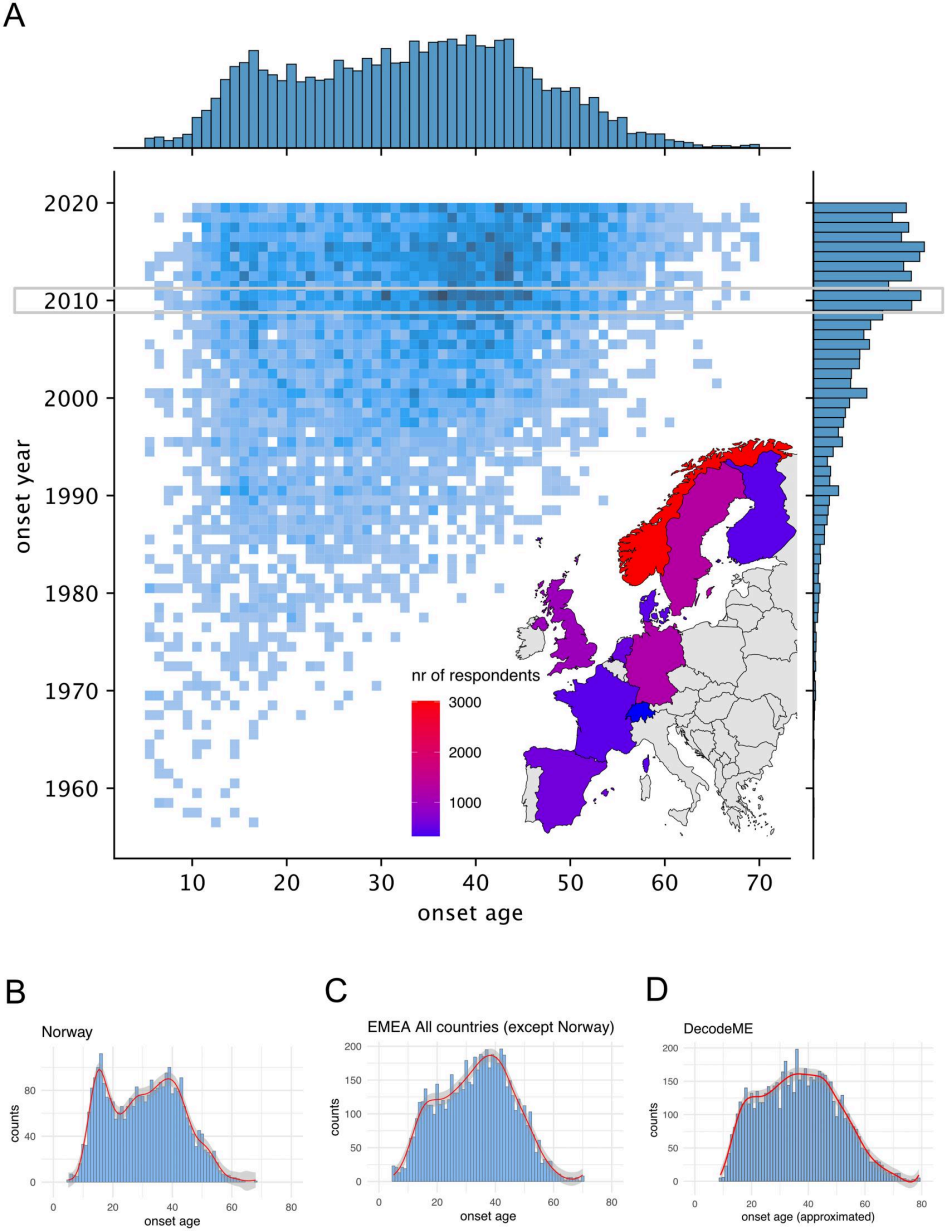
ME/CFS Onset age data over time. (A) Heatmap of incident ME/CFS cases by year and onset age (all countries). Countries included in this study are coloured by number of survey respondents. A potential uptick in ME/CFS cases in 2009/2010 is highlighted with a grey box. (B) ME/CFS onset age distribution for Norwegian respondents, and (C) ME/CFS onset age distribution for respondents from all other countries. (D) Approximated ME/CFS onset age distributions from a subset of DecodeME participants. Onset ages were rounded to the nearest integer to fit the spline. Splines fitted to the data are displayed as red lines and the shaded area represents their standard error.

This study re-analysed a subset of the data collected in the 2021 European ME Association (EMEA) survey [9] from anonymous respondents with ME/CFS from across Europe (*n* = 9380). Due to differences in diagnostic practices in different European countries, we included all respondents with self-reported ME/CFS, regardless of whether they had been diagnosed or not. However, a large majority (89%, Table 1) of respondents indicated that they had received a clinical diagnosis. We included data from the 10 European countries (Table 1) that had an estimated coverage of at least 0.1% of their expected ME/CFS population. Countries’ percentage of responses varied greatly, with the highest fraction of responses in Norway (33.2% of total responses) and the lowest in Switzerland (2.3%) (Fig. 1A, lower right). We further validated our analysis in data from a subset of respondents from DecodeME (*n* = 6455), who self-reported a disease duration of 10 years or fewer, and a trigger of either glandular fever/infectious mononucleosis, other infection, or no infection (Table 1). While exact age of onset was not ascertained in DecodeME data, it was approximated by using the midpoint of each duration bin (0.5–1 years: 0.75 years; 1–3 years: 2 years; 3–5 years: 4 years; and 5–10 years: 7.5 years) and subtracting the midpoint from the age of the respondent at completion of the survey.

**Table 1:** Respondents by dataset, country, and diagnostic status

Dataset	Country	Diagnosed		% Diagnosed	Total
		Yes	Not yet/No		
EMEA	UK	957	31	96.9	988
	Norway	2937	175	94.4	3112
	Netherlands	518	32	94.2	550
	Sweden	1203	121	90.9	1324
	Spain	508	91	84.8	599
	Denmark	391	80	83.0	471
	Switzerland	178	42	80.9	220
	Germany	971	246	79.8	1217
	France	344	113	75.3	457
	Finland	329	113	74.4	442
	Total	8336	1044	88.9	9380
DecodeME subcohort	UK	6455	0	100.0	6455

The average age of respondents who completed the EMEA survey was similar across countries, with an average age of 46.6 years (Supplementary Fig. 1A). The data includes incident ME/CFS cases from as early as 1956. Examining new onset cases by year suggests a peak in 2009 and 2010, coinciding with the 2009 swine flu pandemic [28] (Fig. 1A). This was also reported in another study leveraging Norwegian health records [29]. The distribution of ME/CFS onset age suggests two peaks in incidence, one in adolescence and one in middle age; this pattern does not appear to be due to changes in incidence among particular demographics in specific years (Fig. 1A). The data is more complete from 2000 onwards, which can be explained in part by the data being left truncated: far fewer late onset cases would have been included before the late 1990s or early 2000s.

### Two age peaks in ME/CFS incidence

Given the previously reported bimodal age of diagnosis reported in data from Norway, we analysed multi-modality in onset age in Norway and in other countries. We first used a hypothesis-free approach to examine the shape of the distributions. We fitted a spline to the onset age distribution for Norway, observing a bimodal pattern consistent with that of previous reports [18] (Fig. 1B). Since one third of all responses came from Norway and six countries had fewer than 600 responses (Table 1), we first compared Norway to all other countries to increase our power to detect any bimodal patterns. The combined 9 other countries showed evidence of two peaks in onset age, albeit with a less pronounced early onset peak than the early onset peak in Norway (Fig. 1C). As a sensitivity analysis, we also fitted splines to the onset age distributions of those with and those without a diagnosis, and observed similar bimodal patterns (Supplementary Fig. 1B).

To further validate our findings, we then fitted a spline to the approximated onset age of the DecodeME subcohort. This showed a similar overall pattern to ‘all other countries’ from EMEA (Fig. 1D). The onset age distribution for Norway was then subjected to a Hartigan’s Dip Test [24] which tests the maximum distance between the distribution and its closest theoretical unimodal distribution. This yielded strong evidence for a multimodal distribution (*p* < 2.2 × 10^−16^; Table 2). Similarly, there was significant evidence for multimodality for data from the combined other countries in the EMEA cohort (*p* < 2.2 × 10^−16^), and from DecodeME (*p* < 2.2 × 10^−16^).

**Table 2:** Hartigan’s dip test results.

Country	D statistic	*P*-value
**Norway**	**0.027**	**<2.2 × 10^−16^**
**All other countries (combined)**	**0.016**	**<2.2 × 10^−16^**
**DecodeME subcohort**	**0.015**	**<2.2 × 10^−16^**
All other countries (individual):		
UK	0.023	0.0005
Sweden	0.020	0.001
Germany	0.017	0.012
Finland	0.027	0.021
Spain	0.025	0.012
Denmark	0.026	0.027
*Netherlands*	*0.021*	*0.095*
*France*	*0.020*	*0.28*
*Switzerland*	*0.025*	*0.46*

Countries with non-significant Hartigan’s dip test results are shown in italics.

### Consistent early and late onset age estimates across 10 countries

To probe whether evidence for bimodal onset age replicated in the individual European countries, we performed Hartigan’s Dip Tests for the 9 other countries we included from the EMEA survey. We found that 7/10 countries had statistically significant evidence in the EMEA data of more than one mode for ME/CFS onset age distributions (Table 2). Switzerland, France and the Netherlands data did not yield statistically significant Hartigan’s Dip Test results, likely due to the low number of respondents in Switzerland and France, and an unusually dominant early onset peak in the Netherlands.

**Figure 2:**
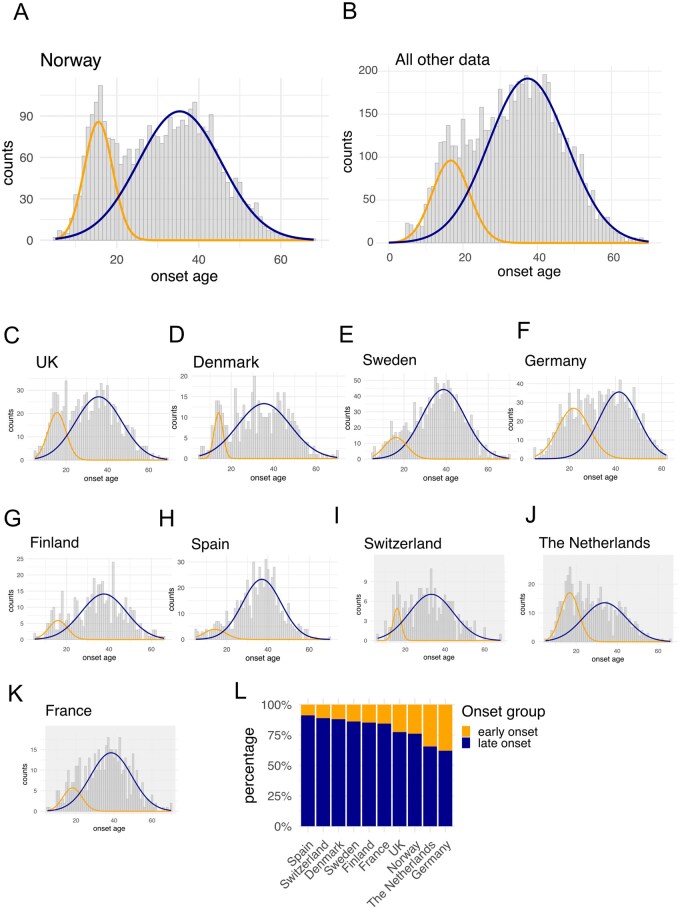
Estimates for early and late onset ME/CFS incidence among European countries. (A) Onset age distributions for Norway and (B) all other countries in EMEA data, overlaid with early- (yellow) and late- (blue) onset distributions modelled in *mixtools* [25]. (C–H) Onset age distributions for all other individual European countries with statistically significant Hartigan’s Dip test results. (I–K) European countries that did not yield statistically significant Hartigan’s Dip test results (i.e. *P* > 0.05). (L) Percentage of data assigned to the early or late onset distributions for each country.

We proceeded to estimate the parameters–two means, two standard deviations, and the relative proportions of the data belonging to each distribution (assuming a Gaussian fit) – of the underlying distributions using Gaussian mixture models. Norway and ‘all other countries’ had similar estimates for mean and standard deviations for both the ‘early onset’ (15.5 and 16.7 years) and ‘late onset’ (35.3 and 37.4 years) distributions (Fig. 2a and b). Early and late onset distributions for the 9 individual other countries yielded remarkably consistent parameter estimates (Fig. 2c–k), as did the DecodeME subcohort, albeit with slightly older estimates for both peaks (Table 3). As a sensitivity analysis, we modelled the data with three Gaussians instead of two. We found that this produced inconsistent results and in many cases one of the three distributions contained < 5% of the data (Supplementary Fig. 2).

**Table 3:** Parameter estimates of onset age distributions using Gaussian mixture models.

Dataset	Country	Mean1	SD1	Percentage1	Mean2	SD2	Percentage2
**EMEA**	Norway	15.5	3.5	23.9	35.3	10.1	76.0
	All other	16.7	5.0	19.2	37.4	10.5	80.8
	UK	15.6	4.3	22.6	35.8	11.2	77.4
	Denmark	14.0	2.0	12.0	35.5	12.4	88.0
	Finland	16.0	4.4	14.8	37.6	10.6	85.2
	Sweden	16.1	5.3	13.8	38.7	10.3	86.2
	Spain	14.1	5.4	8.9	37.0	9.4	91.1
	Germany	21.8	6.8	37.9	41.5	8.5	62.1
	*Switzerland*	*15.8*	*2.0*	*11.1*	*33.1*	*11.0*	*88.9*
	*Netherlands*	*16.8*	*4.4*	*34.4*	*33.9*	*10.6*	*65.6*
	*France*	*18.1*	*5.0*	*15.6*	*38.5*	*10.8*	*84.4*
**DecodeME subcohort**	UK	18.8	4.3	17.1	40.1	12.2	82.9

Percentage: percentage of the data fitted to first or second distributions. Countries with non-significant Hartigan’s dip test results are shown in italics.

However, the proportions of data modelled as part of early or late onset distributions varied considerably across countries (Fig. 2l). Germany had the latest early onset peak, which was also the broadest (average age 21.8 years, 37.9% data; Table 3), and Spain the least pronounced early onset peak (8.9% data). The UK and Norway had the most similar onset age distributions in terms of these parameter estimates and the relative proportions of the early and late onset peaks. The Hartigan’s Dip Test results, the consistency of the parameter estimates from the Gaussian mixture models, and the replication between EMEA and the subset of the DecodeME cohort provide consistent and strong evidence for a bimodal distribution of ME/CFS onset age.

Survey data is subject to reporting and sampling biases [30, 31], and we considered the possibility that our results could be affected by the data truncation in the EMEA dataset evident from Fig. 1A. Furthermore, our analyses did not account for population age structures. We performed several sensitivity analyses to confirm that the observed bimodal age at onset distribution is robust to these effects. We subsetted the data to include only onset cases between 2011 and 2020 (Supplementary Fig. 3A; Supplementary Tables 1 and 2) to account for the data truncation, finding similar results to the primary analyses. Additionally, since there is demographic data available from this period, we adjusted the incidence by age group in the EMEA data for Europe-wide population age structure (Supplementary Fig. 3B). We also performed a similar analysis accounting for UK population age structure across all ages in both UK-only EMEA and DecodeME datasets (Supplementary Fig. 3C and 3D). We find that our results hold, and by accounting for population age structure in recent years the early peak becomes more pronounced.

### Glandular fever/infectious mononucleosis is a common trigger in early onset ME/CFS

**Figure 3:**
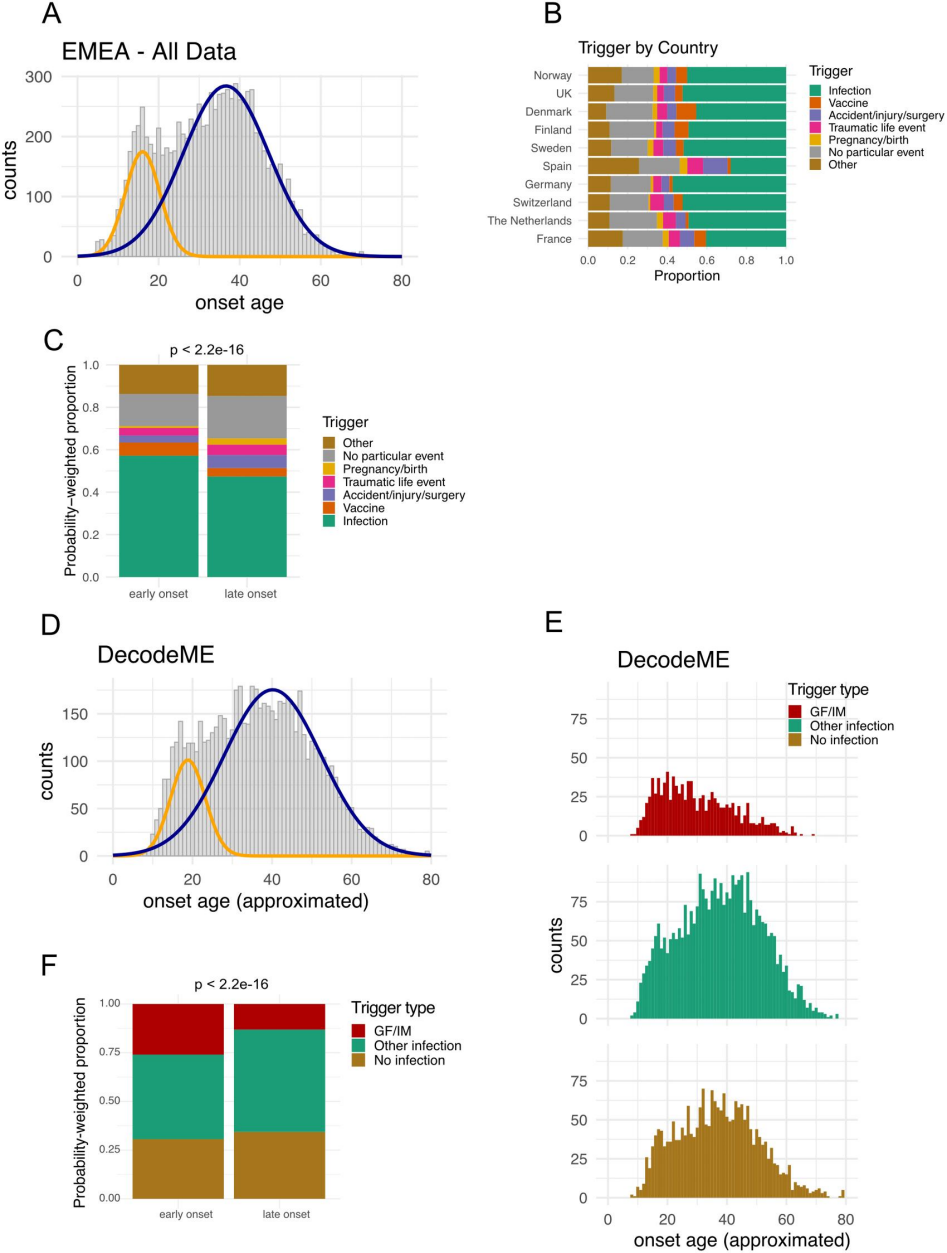
Trigger types in early and late onset ME/CFS. (A) Age of onset data with early onset (yellow), or late onset (blue) distributions fitted to the combined data from all countries using Gaussian mixture models, used to assign respondents a probability of belonging to early and late onset groups for subsequent analyses. (B) Proportion of respondents reporting each trigger, by country. (C) Probability-weighted proportion of data assigned to either early or late onset distributions by trigger type. (D) Early onset (yellow), or late onset (blue), distributions fitted to DecodeME subcohort data using Gaussian mixture models, used to assign respondents a probability of belonging to early or late onset groups for subsequent analyses. (E) DecodeME subcohort onset age distributions shown for the three trigger types considered in this analysis: glandular fever/infectious mononucleosis (GF/IM), ‘Other infection’, or ‘No infection’. (F) Probability-weighted proportion of data assigned to either early or late onset distributions by trigger type in DecodeME subcohort data. P-value was obtained from a Pearson’s Chi-squared test comparing the frequencies of trigger types between early and late onset distributions.

We next considered whether there were explanatory variables that differ between early and late onset ME/CFS that might indicate underlying biological mechanisms. Therefore, we compared key characteristics between the two peaks. Due to the low number of respondents in some countries, we analysed the combined data from all 10 countries in the EMEA data to increase our statistical power. We assigned to each datapoint the probabilities of belonging to the early (mean 1 : 16.0 years, SD 1 : 4.3) or the late onset (mean 2 : 36.6 years, SD 2 : 10.5) distribution (Fig. 3a).

We first hypothesised that early and late onset disease may be associated with different triggers. Proportions of triggers were consistent across different countries (Fig. 3b) with the sole exception of Spain, which had a markedly low proportion of respondents reporting infectious triggers and an increased proportion of respondents reporting ‘Other’ or ‘Accident/injury/surgery’ as triggers. Infection was the dominant trigger in both the early (57%) and late onset ME/CFS groups (47%). The frequency of trigger types was significantly different between the early and late onset groups (Chi-squared = 117.6, df = 6, *p* < 2.2 × 10^−16^). Examining the residuals, there was a larger relative frequency of respondents in the early onset ME/CFS group who reported an infectious trigger than in the late onset group (Bonferroni-adjusted *p* = 2.1 × 10^−13^) (green proportion in Fig. 3c). The same was true for those who reported vaccination as a trigger (Bonferroni-adjusted *p* = 0.0002). Conversely, ‘Accident/injury/surgery’ (Bonferroni-adjusted *p* = 1.6 × 10^−5^), ‘No particular event’ (Bonferroni-adjusted *p* = 1.7 × 10^−5^), and ‘Pregnancy/birth’ (Bonferroni-adjusted *p* = 3.3 × 10^−6^) were more frequent triggers in the late onset group. There was no statistically significant difference in frequency of ‘Traumatic life event’ or ‘Other’ as triggers between the two onset age groups (Bonferroni-adjusted *p* > 0.05). However, all triggers appeared to have both early and late onset cases (Supplementary Fig. 4).

We subsequently performed the same analysis for DecodeME survey responses (Fig. 3d) and stratified respondents by trigger type. While the ‘Other infection’ and ‘No infection’ categories had similar onset age profiles (Fig. 3e), the distribution for the glandular fever/infectious mononucleosis category was right-skewed. The frequency of trigger types was significantly different between the early and late onset groups (chi-squared = 117.37, df = 2, *p*< 2.2 × 10^−16^). Those reporting glandular fever/infectious mononucleosis as a trigger had greater odds of having early onset ME/CFS relative to those who reported ‘Other infection’ or ‘No infection’ as a trigger (Fig. 3f) (OR 2.32, 95% CI [1.99–2.71], *p* = 2.4 × 10^−24^).

In summary, infectious onset, in particular glandular fever/infectious mononucleosis, occurred more frequently among those with early onset disease.

### Greater burden of severity in early onset ME/CFS

**Figure 4.**
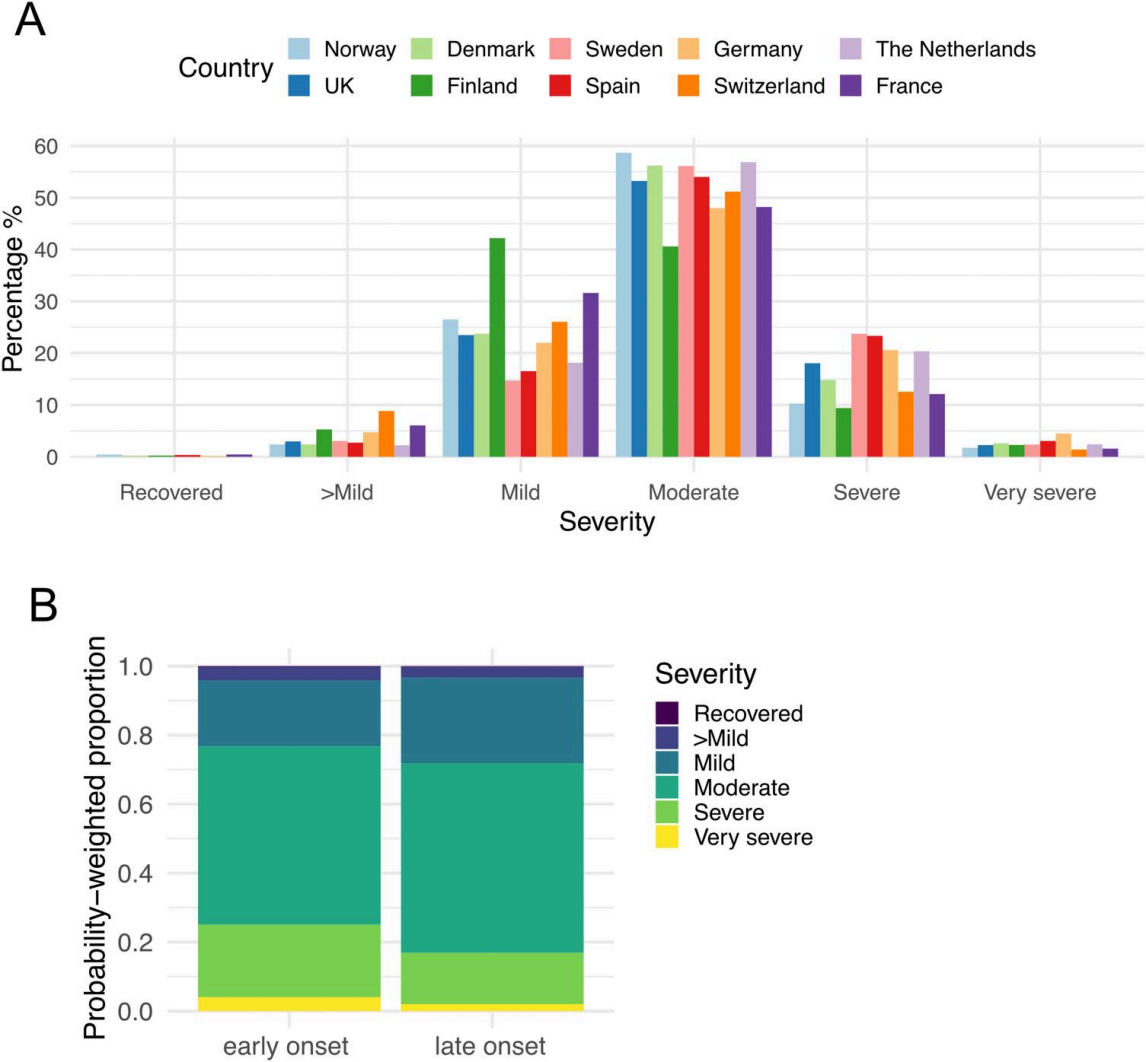
Disease severity in early and late onset ME/CFS. (A) Breakdown of severity groups for each country. (B) Probability-weighted proportions of each severity group in early or late onset ME/CFS.

Next, we explored whether severity differed in early versus late onset ME/CFS. Severity was assessed according to the International Consensus Criteria definitions: Mild (approximate 50% reduction in pre-illness activity level), moderate (mostly housebound), severe (mostly bedridden) or very severe (totally bedridden and needs help with basic functions) [32], with the additional categories of ‘Better-than-mild’ and ‘Recovered’. Across all countries, about half of all respondents reported moderate ME/CFS (range: 40.6%–58.7%) (Fig. 4a). This was the majority severity category, except for Finland where slightly more respondents had mild than moderate ME/CFS (42.2% versus 40.6%). The percentage of severely affected individuals varied by country with Sweden and Spain having the highest (23.7% and 23.3%) and Finland the lowest percentage of severely affected respondents (9.4%). Testing the association between ME/CFS severity and onset group using a non-proportional odds ordinal logistic regression revealed that early onset ME/CFS was associated with more severe disease, while the late onset group was more strongly associated with moderate and mild disease (Fig. 4b, Supplementary Table 1). The odds of being moderate, severe or very severely affected compared to mild, better than mild or recovered were greater for the early onset group with an odds ratio of 1.4 (95% CI [1.20–1.63], *p* = 1.83 × 10^−5^). Similarly, the odds of being severe or very severely affected, compared to moderate or milder, were greater for early relative to late onset ME/CFS with an odds ratio of 2.15 (95% CI [1.84–2.51], *p* < 2 × 10^−16^). The effect was even larger when comparing the very severely affected to those who were severe or milder, with greater odds of being very severe for those in the early onset ME/CFS group (OR: 3.33, 95% CI [2.31–4.81], *p* = 1.18 × 10^−10^).

We simultaneously tested whether disease duration was associated with increased severity in early onset ME/CFS in our data. Illness duration was associated with slightly decreased odds of being more severe per year of illness. This was significant only when comparing recovered, >mild or mild ME/CFS, to moderate, severe, or very severe disease, with a very modest effect (OR = 0.992, 95% CI [0.988–0.997], *p* = 0.0002). We observed the opposite direction of effect for duration on severity when comparing very severe ME/CFS to severe or milder (1.019, 95% CI [1.004–1.032], *p* = 0.01). Results for all comparisons between severity categories for duration and onset group are provided in Supplementary Table 3. The variance inflation factor was very low (VIF: 1.06) indicating that multicollinearity between duration and onset group was negligible. These results suggest that illness duration is only modestly associated with severity, and this effect is subtle because we expected a consistent direction of effect across groups of increasing severity. Thus, the increased odds for severe or very severe disease in those with early onset ME/CFS appear *not* primarily to be due to illness duration.

### Comparable ratio of male to female ME/CFS cases in early and late onset peaks

**Figure 5:**
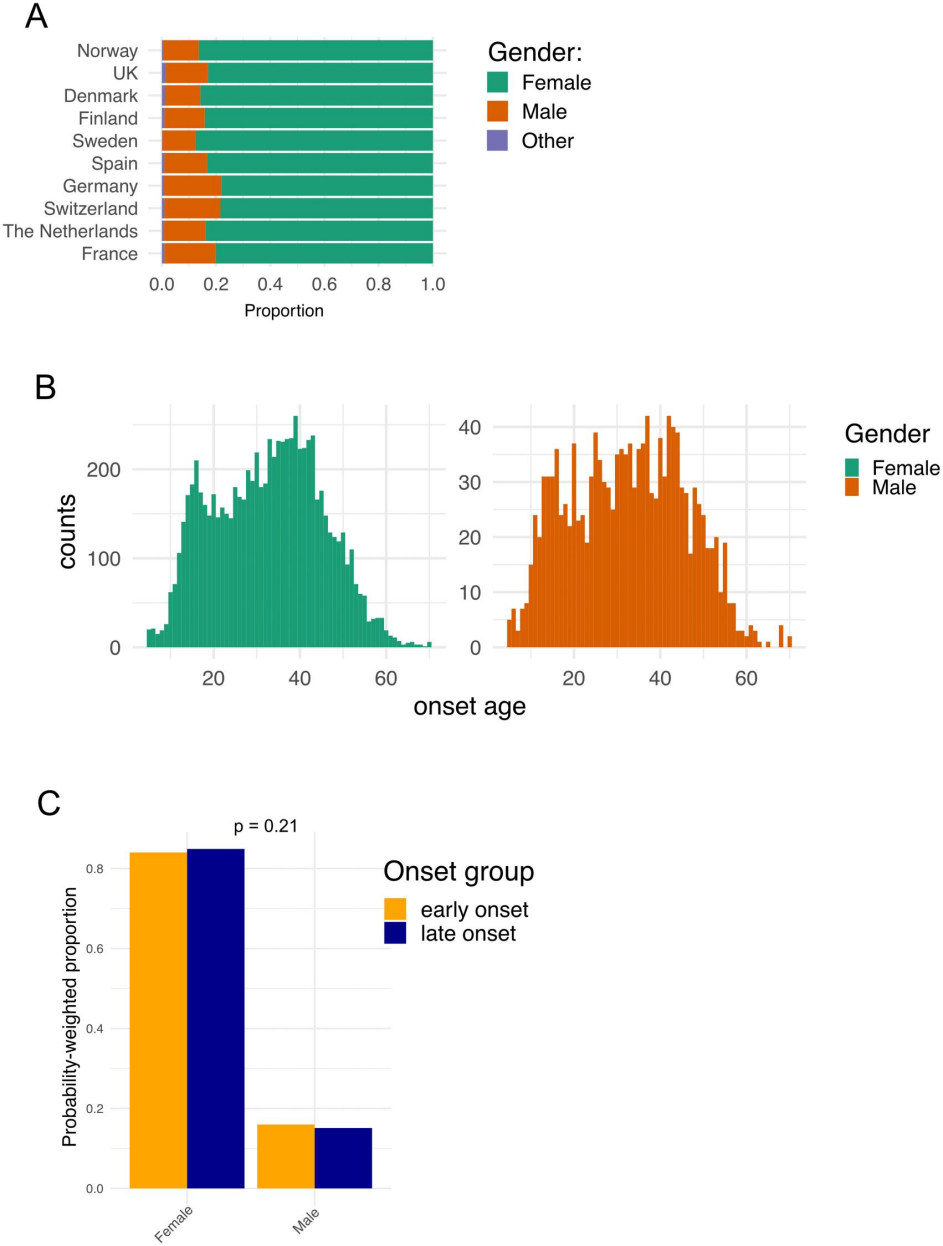
Gender ratios for early and late onset ME/CFS. (A) Proportion of respondents in each country of male, female or ‘other’ gender. (B) Histograms for age at onset for females and males for all EMEA data. (C) Proportion of data for female or male gender in early or late onset distributions. P-value was derived from Pearson's Chi-squared test comparing frequencies of genders between early and late onset distributions, alpha = 0.05.

Bakken *et al*. (2014), reported a higher proportion of females diagnosed in the second age peak. We therefore hypothesised that the composition of the early and late onset peaks may differ by gender. Nevertheless, gender proportions were consistent across countries, at 78%–86% female (Fig. 5a and b). There was no significant difference in the probability-weighted frequencies of male and female genders in the early and late onset groups (Chi-squared = 1.59, df = 1, *p*= 0.21) (Fig. 5c).

### Having a relative with ME/CFS increases odds of early onset disease

**Figure 6:**
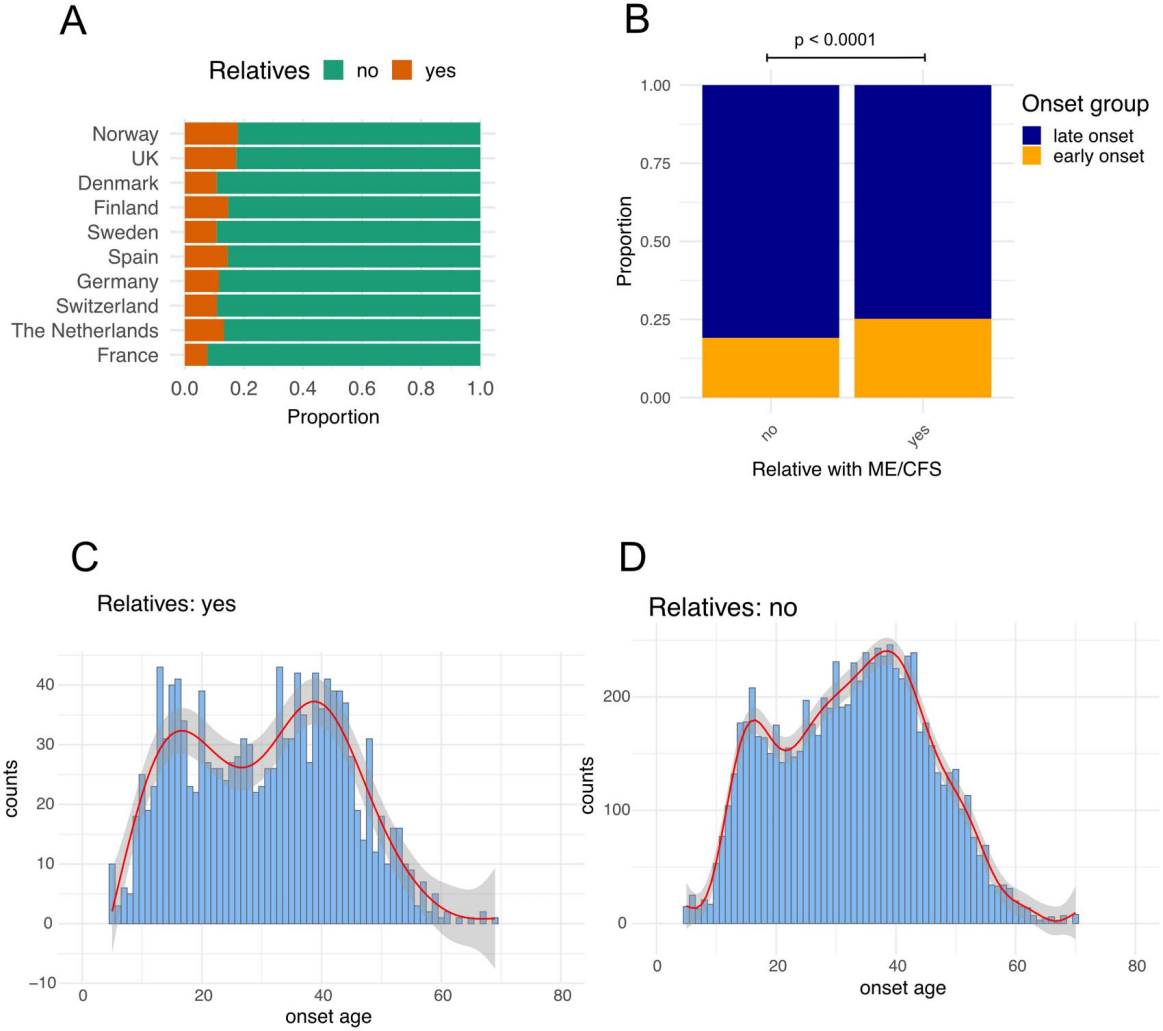
Individuals with first-degree relatives with ME/CFS in early and late onset ME/CFS. (A) Proportion of respondents in each country with, or without, first-degree relatives with ME/CFS. (B) Probability-weighted proportions of early or late onset ME/CFS with and without first-degree relatives with ME/CFS. *P*-value derived from Fisher’s exact test for odds ratio calculated from a 2 × 2 contingency table. (C) Onset age distributions for those with first-degree relative with ME/CFS, and (D) those with no first-degree relative with ME/CFS. Fitted splines are displayed as red lines with the shaded area indicating their standard error.

To explore potential differences in genetic and/or environmental risk between early or late onset ME/CFS, we asked whether having a relative with ME/CFS was more common in either the early or late onset age groups. We hypothesised that cases with first degree relatives with ME/CFS were more likely to harbour greater risk for developing ME/CFS due to shared genetics or shared environmental exposures. On average 13.0% of respondents had a first degree relative with ME/CFS, with Norway reporting the highest percentage of relatives (17.9%), and France the lowest (7.7%) (Fig. 6a). A larger percentage of respondents in the early onset group had one or more first degree relatives with ME/CFS (22.5%) relative to the later onset group (14.9%) (Fig. 6b and d). Those with relatives with ME/CFS were more likely to belong to the early onset group, with an odds ratio of 1.43 (95% CI [1.25–1.63], *p* = 4.4 × 10^−07^), which is considered a moderate effect size.

## Discussion

Hospital or outpatient-diagnosed cases in Norway show two peaks for incidence age of ME/CFS [18]. Here, we generalised this finding to other European countries, and replicated it using larger samples in two datasets. Early and late onset peaks were both evident in most countries, and were strikingly consistent across countries with respect to their means and standard deviations. Onset-age distributions from a DecodeME subcohort further supported this observation. DecodeME estimates for both peaks were slightly older, possibly because this study only recruited participants aged 16 and older, and because onset age was derived from duration bins with an error of ± 2.5 years. Importantly, only approximately 10% of DecodeME respondents were aged 30 or younger [10] and since respondents included in this subcohort needed to have a disease duration of <10 to enable us to approximate onset age, early onset cases are likely to be under-represented in this cohort. Previous work exploring onset age patterns in ME/CFS used either large-scale diagnostic or health insurance claims data [11, 18, 20, 33–35], or small-scale surveys [36–39]. Publicly available summary data from the Finnish patient registry provides further support for a bimodal distribution based on age at first entry of the G93.3 ICD10 code (*n* = 1622) [40]. A key strength of our study is that it is the first to examine age at onset (rather than diagnosis) in a large dataset covering multiple countries with diverse healthcare systems. Since substantial ME/CFS diagnostic delays and inequalities are unfortunately widespread [9, 11], our onset-age estimates capture the incidence of ME/CFS across different age groups more accurately.

A limitation of our study is that surveys collecting self-reported health outcomes are likely to suffer from ascertainment and recall biases. Ascertainment biases might have resulted in under-reporting of ME/CFS onset among older adults who are less likely to engage in online surveys [30, 31]. Furthermore, since only respondents who were alive in 2021 could complete the EMEA survey, cases with a long disease duration by necessity had onset at a younger age. This means that our parameter estimates for the late-onset peak may be biased towards a younger average as a result. Recall biases could affect both the parameter estimates for age at onset, and the reported trigger types [41]. A further limitation of the EMEA dataset is that diagnostic practices for ME/CFS vary across Europe [42], and we did not require respondents to report a diagnosis. It is possible that some respondents in the EMEA dataset would not meet CCC or IoM diagnostic criteria. However, these limitations were addressed by replicating our findings in a DecodeME subcohort. In the DecodeME subcohort we only analysed data from individuals who met CCC or IoM diagnostic criteria and who had a disease duration of 10 years or less, and we observed results consistent with EMEA. DecodeME participants’ ages are similar to England-wide patients with electronic healthcare record links to G93.3 (Postviral fatigue syndrome), except for a slight under-representation of older individuals aged 70 and over [10]. Furthermore, our sensitivity analyses (Supplementary Fig. 3) demonstrated that even when only considering onset cases between 2011 and 2020 in the EMEA dataset, the age at onset distribution remained bimodal, and the early onset peak was even more pronounced when accounting for population age structure.

Comparing trigger types between early and late onset cases revealed that infection, particularly glandular fever/infectious mononucleosis, was more common in early onset disease. Those with early onset ME/CFS were more likely (OR: 2.32) to report glandular fever/infectious mononucleosis as a trigger, than those who reported ‘Other infection’ or ‘No infection’ in DecodeME. Notably, the age profile for early onset ME/CFS in the UK in this dataset is similar to that for glandular fever/infectious mononucleosis diagnoses in the UK, with most cases occurring in the 15–19 age group [43].

Furthermore, while the incidence of glandular fever/infectious mononucleosis is 5 per 1000 in the UK [44], it is an order of magnitude smaller (0.4 per 1000) in Spain [45], which in our dataset had the smallest early onset peak. Vaccines against Epstein-Barr virus, which causes most glandular fever/infectious mononucleosis cases, are not yet available but are being developed [46, 47], and could in future have a potentially substantial impact on reducing ME/CFS case burden, particularly for early onset disease.

One possibility is that late onset ME/CFS might arise from a more heterogeneous range or accumulation of triggers, supported by the relative increase in cases in the late onset group who reported ‘No particular event’ as a trigger and the larger standard deviation of the late onset peak. Hormonal changes associated with puberty and peri-menopause may also be implicated [48], although we might expect to see stronger sex differences in incidence age if this were the case. Future studies that ascertain both ME/CFS onset age and triggers in greater detail, including which infections are implicated and the severity of these infections, could help to resolve these questions.

Bimodal onset age patterns, although uncommon, have been observed for other diseases [16, 49–52]. Among these diseases, the age profile of ME/CFS is unique. In an analysis of the incidence of 19 autoimmune diseases across >22 million GP records in the UK, coeliac disease, inflammatory bowel disease, and vasculitis each had unique bimodal age at diagnosis distributions, yet none of these corresponded to the age peaks observed in ME/CFS [49].

Although there was statistical support for two onset age peaks in most countries, the relative proportion of people with early or late onset ME/CFS differed substantially between countries. The UK showed the most similar onset age profile to Norway. Despite their geographical proximity, Sweden had an early onset peak that was notably smaller than the Norwegian peak. Sweden has adopted a predominantly psychological model of ME/CFS [53, 54]. Consequently, the inter-country variation may be partially explained by differing societal attitudes towards and awareness of ME/CFS, which could affect ascertainment biases. In countries with better awareness of the condition and more available services, young people with ME/CFS (and their parents) may be more likely to hear about the condition, receive a diagnosis, and/or follow the ME/CFS non-profit or support groups that advertised the EMEA survey. It is also possible that different environmental and/or pathogen exposures might influence the relative size of the early onset peak. While we cannot rule out the contribution of non-biological factors to the bimodal onset pattern observed in this study, comparable observations from EHR data suggest that there are likely to be biological factors contributing to the bimodal onset age of ME/CFS.

While further work is needed to fully understand ME/CFS subgroups, stratifying cases based on onset age in future research may help to reduce cohort heterogeneity and increase the power to detect biological differences. Such approaches may help uncover disease mechanisms and reveal possible preventative measures, such as vaccination against ME/CFS-associated pathogens.

## Supplementary Material

iqag007_Supplementary_Data

## Data Availability

No new data was generated for this study. The data in this study can be requested from the European ME Alliance (https://www.euro-me.org/index.shtml), and the DecodeME project (https://institute-genetics-cancer.ed.ac.uk/decodeme-the-worlds-largest-mecfs-study/researcher-access).
