# Supplementary material for: Incidence age is bimodal for myalgic encephalomyelitis/chronic fatigue syndrome, with higher severity burden for early onset disease
Source: Oxf Open Immunol. 2026 Mar 17;7(1):iqag007. doi: 10.1093/oxfimm/iqag007 (PMC13070794; doi:10.1093/oxfimm/iqag007)
Supplement: iqag007_Supplementary_Data [file iqag007_supplementary_data.zip › 18-Mar-2026_053810_Supplementary_material.docx]

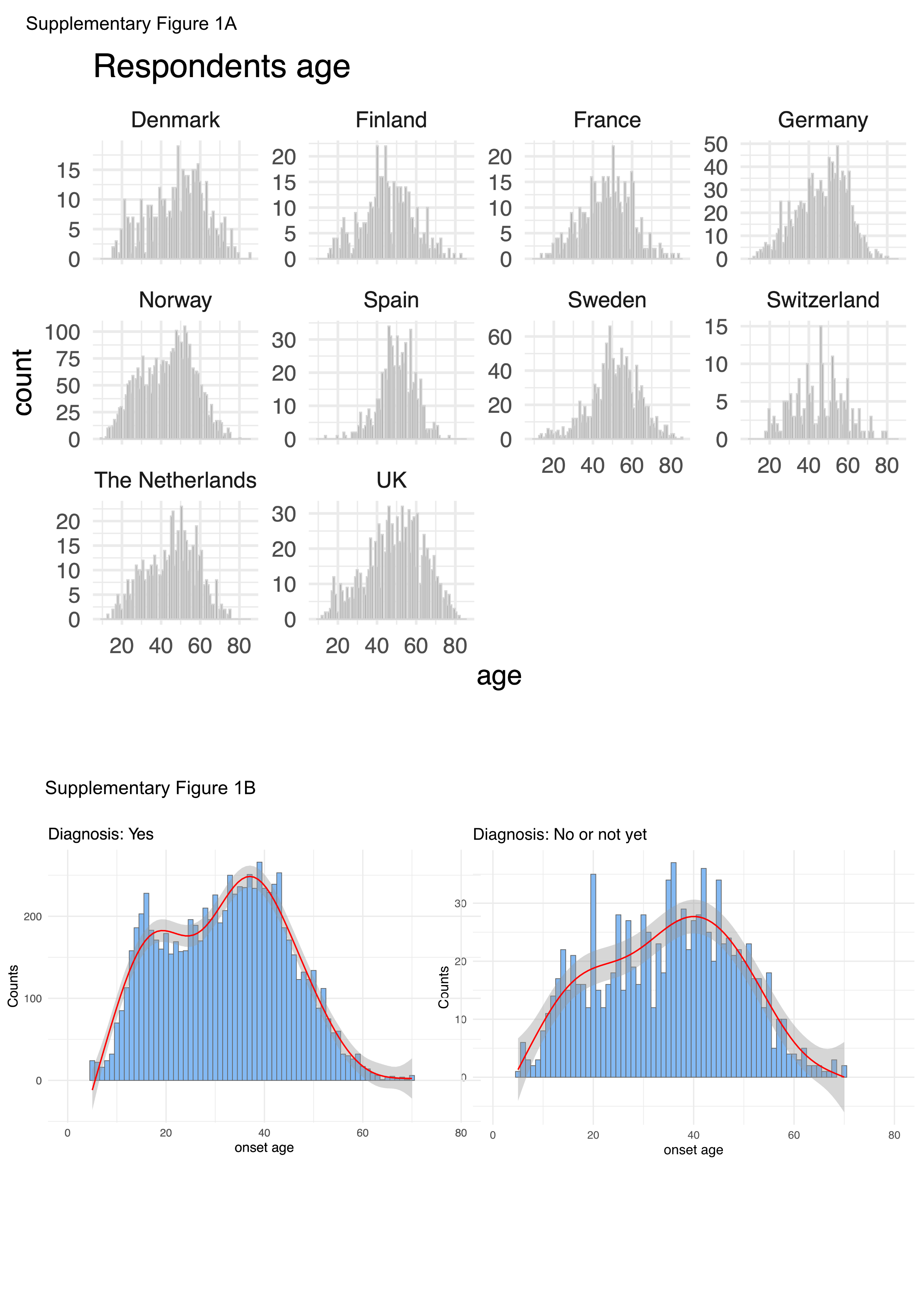


**Supplementary Figure 1:** A) Age distribution of respondents by country. B) Age at onset distributions with fitted splines for individuals reporting a diagnosis or no diagnosis/not yet diagnosed.


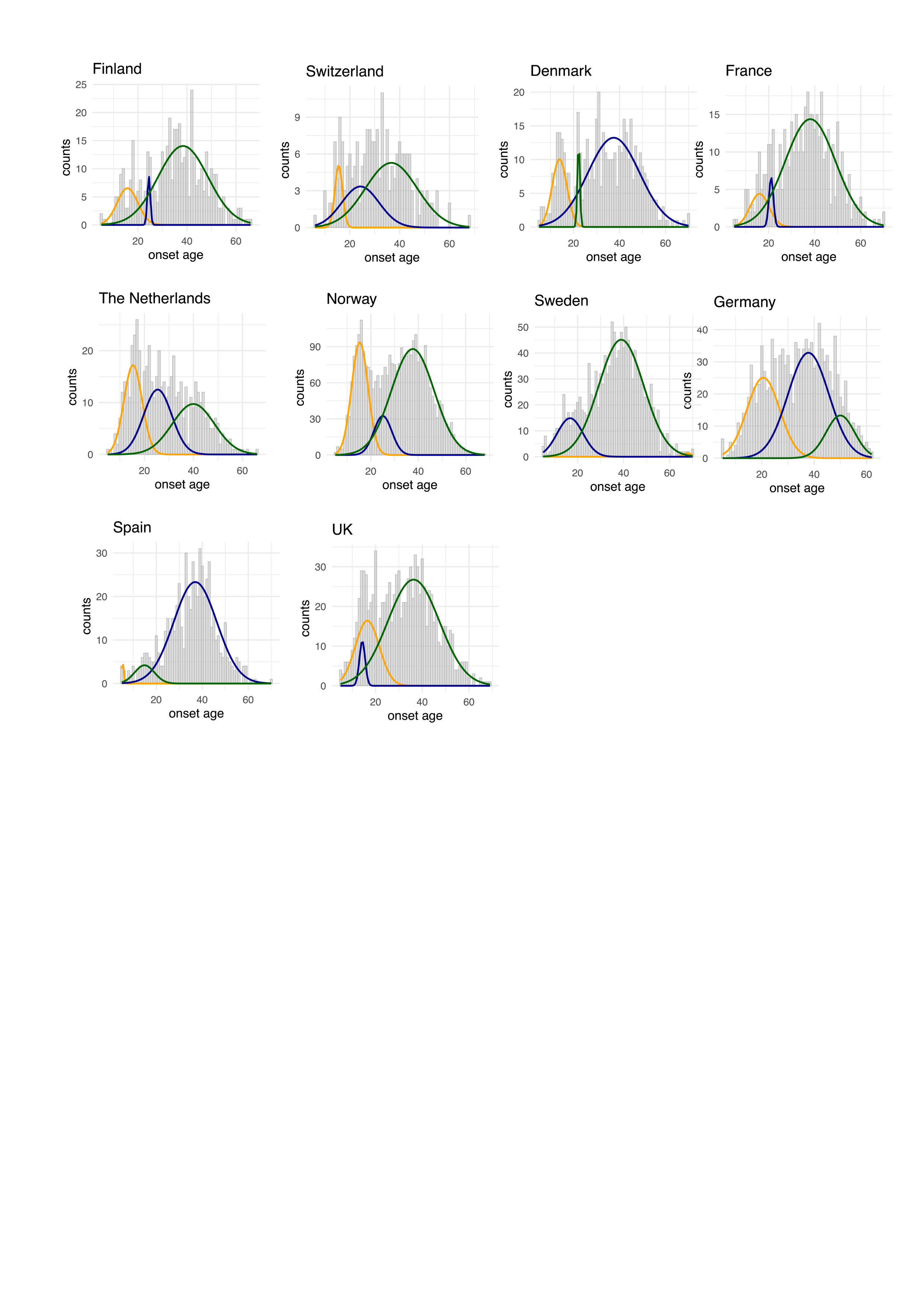


**Supplementary Figure 2: Onset age distributions for all countries in EMEA data, overlaid with 3 Gaussian distributions modelled in *mixtools.***

Model fits with the greatest log likelihood were selected from 1000 iterations.


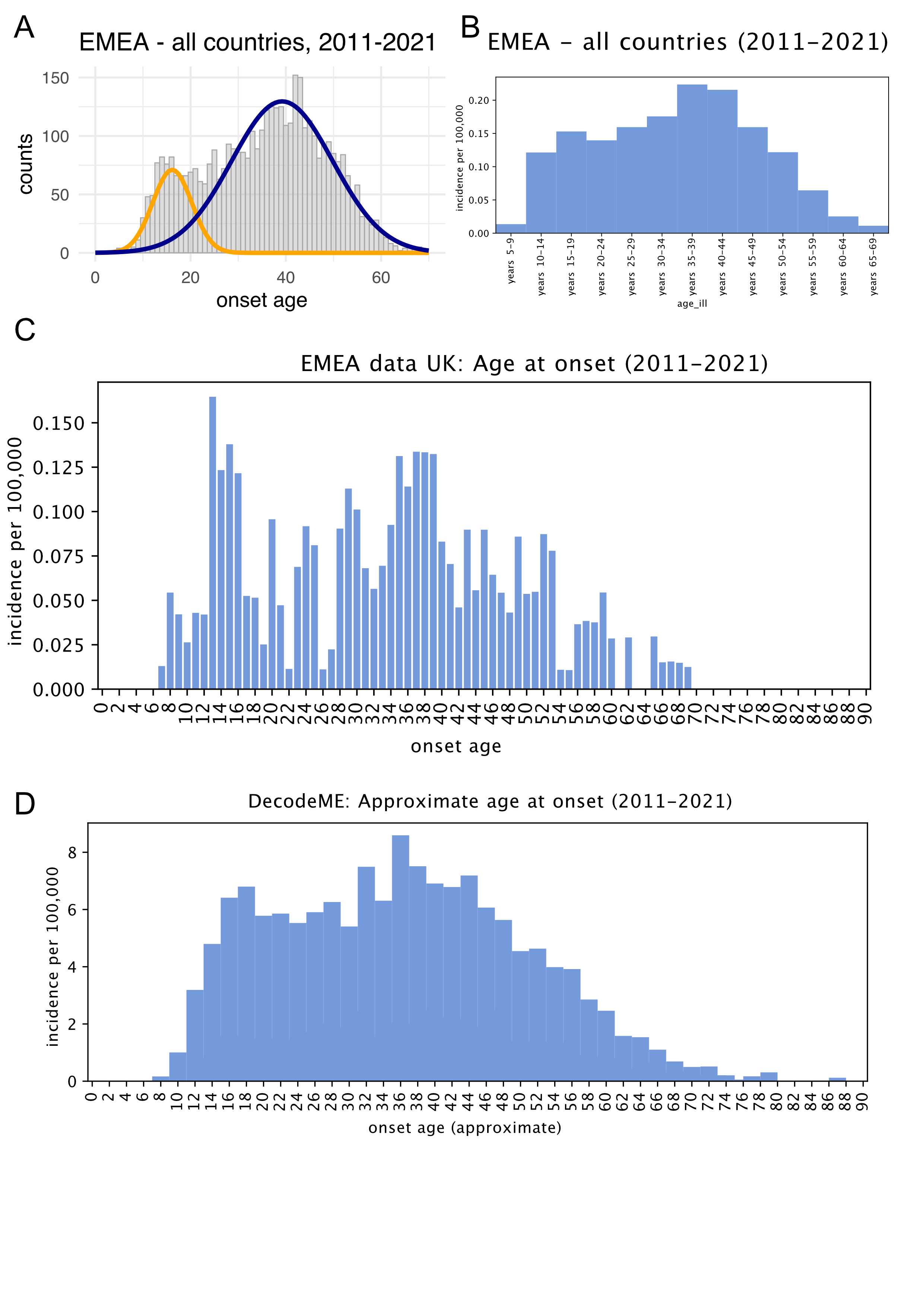


**Supplementary Figure 3:** A) Two Gaussian distributions fit to EMEA data subset to onset cases between 2011-2021. B) Incidence estimates for each age group adjusted for the population age structure for Europe from 2011-2021. Data source Eurostat: <https://ec.europa.eu/eurostat/web/population-demography/demography-population-stock-balance/database>. C) Incidence estimates for each age based on EMEA UK data, subset to onset cases between 2011-2021, adjusted for the population age structure for UK from 2011-2021. Annual incidence per age averaged across years. Data source ONS (Population by single year of age and sex, England, Wales, Scotland and Northern Ireland, mid-2011 to mid-2024): <https://www.ons.gov.uk/peoplepopulationandcommunity/populationandmigration/populationestimates/bulletins/annualmidyearpopulationestimates/latest#age-structure-of-the-population>. D) Incidence estimates for each age based on subset of DecodeME participants (as shown in Figure 3D), adjusted for the population age structure for UK from 2011-2021, annual incidence per age averaged across years.


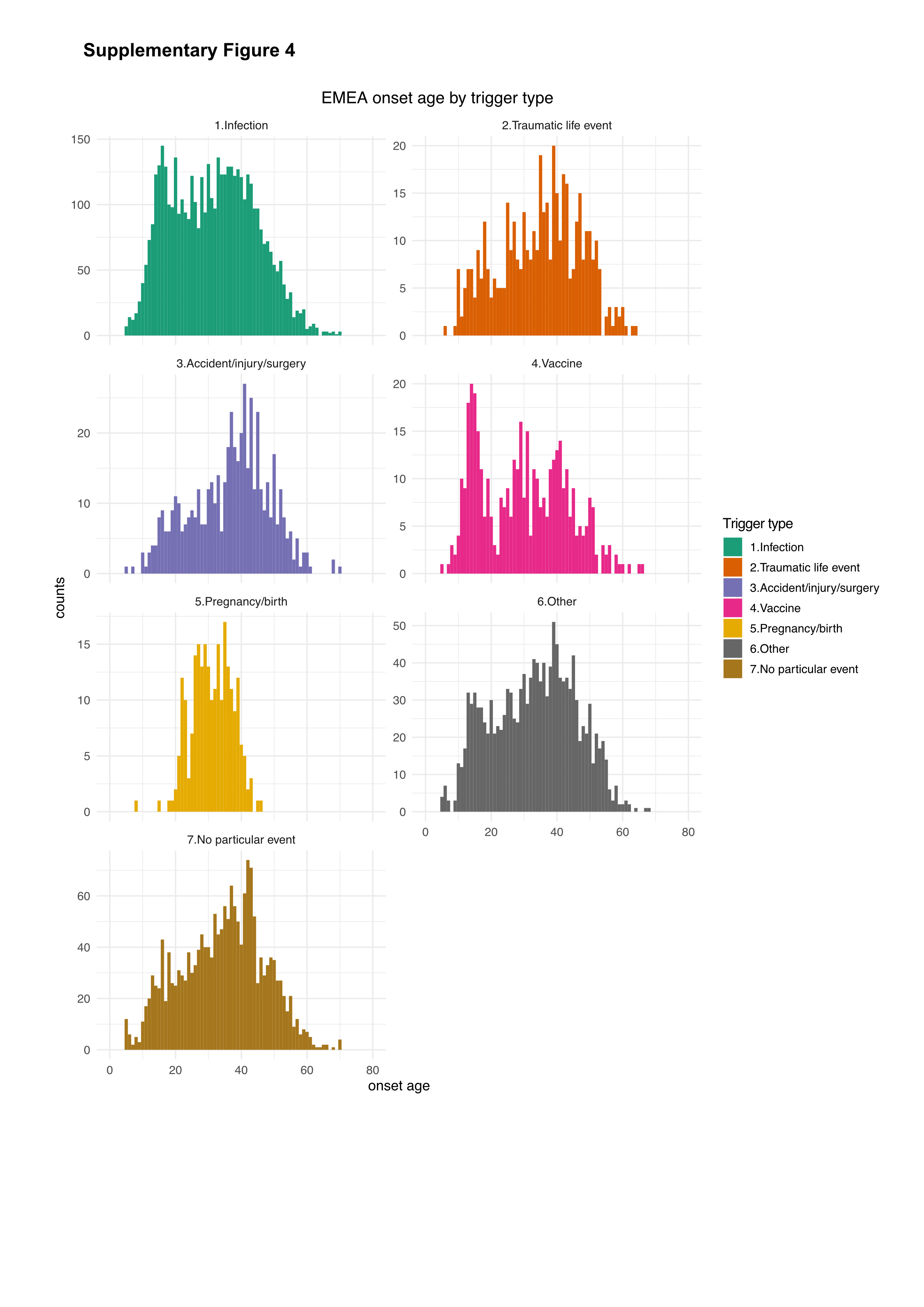


**Supplementary Figure 4: Age at onset by trigger type.**


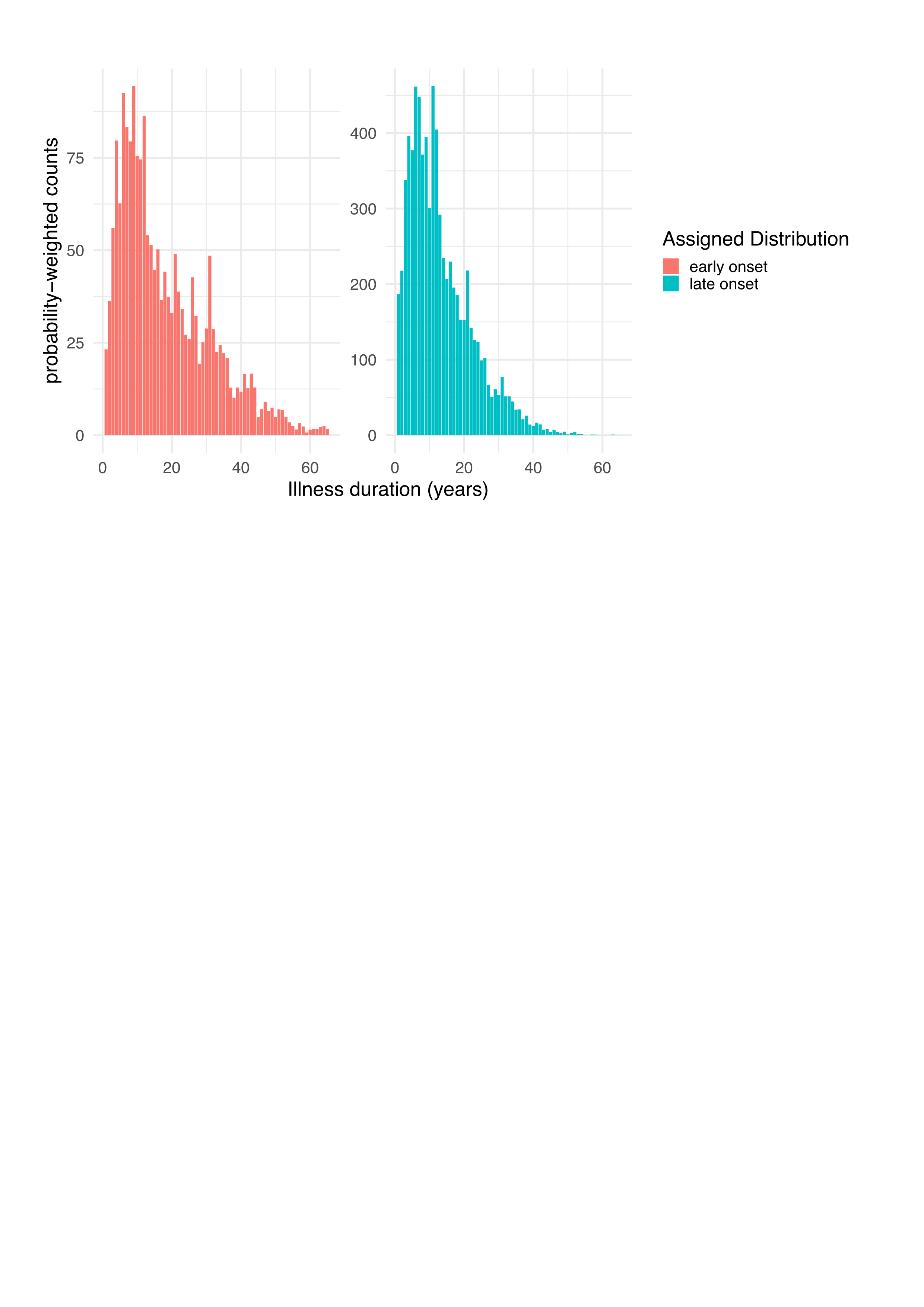


**Supplementary Figure 5: Illness duration for early and late-onset ME/CFS**

**Supplementary Table 1**

| EMEA data 2011-2021, Hartigan’s Dip Test: | |
| --- | --- |
| D = 0.018 | P value < 2.2x10^-16^ |

**Supplementary Table 2:**

| EMEA data 2011-2021, parameter estimates from mixture model | | |
| --- | --- | --- |
|  | **Early onset** | **Late onset** |
| **mean** | 16.1 | 39.2 |
| **standard deviation** | 4.1 | 10.6 |
| **percentage** | 17.5% | 82.5% |

**Supplementary Table 3: vglm coefficients with 95% CI (exponentiated)**

From the model:

Severity ~ duration + probability_late

|  | duration (exponentiated) | p value (unadjusted) | Onset group (exponentiated) | p value (unadjusted) |
| --- | --- | --- | --- | --- |
| Recovered vs >mild, mild, moderate,  severe or very severe | 0.968 [0.920-1.018] | 0.20917 | 0.729 [0.196-2.721] | 0.63861 |
| Recovered  or >mild vs mild, moderate, severe or very severe | 1.005 [0.995-1.016] | 0.31367 | 0.755 [0.538-1.059] | 0.10381 |
| Recovered,  >mild or mild, vs moderate, severe or very severe | 0.992 [0.988-0.997] | 0.00204 | 1.396 [1.198-1.628] | 1.93E-05 |
| Recovered,  > mild, mild or moderate vs severe or very severe | 0.998 [0.993-1.003] | 0.46495 | 2.149 [1.835-2.516] | <2 x 10E-16 |
| Recovered,  > mild, mild, moderate or severe vs very severe. | 1.018 [1.004-1.032] | 0.01012 | 3.334 [2.311-4.810] | 1.18E-10 |
